# 287 Assessment of the National Burn Repository’s Ability to Examine Obesity and Determine Burn Related Outcomes

**DOI:** 10.1093/jbcr/irad045.262

**Published:** 2023-08-29

**Authors:** Edward J Kelly, Lauren T Moffatt, Jeffrey W Shupp, Shawn Tejiram

**Affiliations:** Firefighter’s Burn and Surgical Research Laboratory, Washington, District of Columbia; Medstar Health, Washington DC, District of Columbia; Firefighters’ Burn and Surgical Research Laboratory, MedStar Health Research Institute, Georgetown Univeristy School of Medicine, Washington, District of Columbia; MedStar Washington Hospital Center, Washington, District of Columbia

## Abstract

**Introduction:**

Despite obesity being a national epidemic there is a paucity of literature examining the effect of obesity in burn-injured patients. Controversy exists on whether obesity provides a protective benefit dubbed the “Obesity Paradox”. Previous literature examining the role of the National Burn Repository (NBR) in obesity-related research identified a lack of nutrition-related fields as a limitation of the dataset. The aim of this work was to provide an interval assessment of the NBR dataset and determine if an effect of obesity on burn-related outcomes and care can be elucidated.

**Methods:**

A retrospective review of patient data contained within the NBR dataset from 2016-2018 was conducted. The data dictionary was queried for patients with “obesity” listed as a comorbidity. Information on patient weight was available, but no data on height, body mass index, or other nutrition-related fields were found. The primary outcome measure was mortality. Secondary outcomes included hospital length of stay (LOS), ICU LOS and total hospital costs. Patients were stratified by total body surface area burn (TBSA) to examine the effect of obesity on mortality under different injury severities. Mann-Whitney Tests were used to compare obese and non-obese patients. Multiple logistic regression (MLG) was used to compare effects of several common independent variables (age, gender, inhalation injury (IHI), percent full thickness burn (%FT), TBSA and obesity) on mortality, ICU LOS >7days, hospital LOS >10 days and total hospital costs >$200,000.

**Results:**

Of 41,031 patients included in the analysis, 3,845 (9.37%) were identified as obese. Overall, obese patients had a higher mean TBSA (8.68 ± 13.19% vs. 7.83 ± 12.39%, p=0.01), but not a higher %FT (7.86 ± 14.09% vs. 7.60 ± 14.25%, p=0.85) or occurrence of inhalation injury (8.92 vs. 8.86%, p=0.90). Obese patients had significantly longer hospital LOS (11.93 ± 18.92 days vs. 10.13 ± 18.73 days, p< 0.001), ICU LOS (10.03 ± 18.96 days vs. 9.44 ± 17.68 days, p< 0.001) and total hospital costs ($364,385 vs $211,513, p< 0.001). MLG found obesity to an independent predictor of ICU LOS >7 days, hospital LOS >10 days and total hospital costs >$200,000 (p=0.036, p=0.039 and p=0.046, respectively). MLG also identified age, TBSA and %FT as independent predictors of mortality in the < 20% TBSA group. Age, TBSA, %FT and IHI were independent predictors of mortality in the 20-40% and >40% TBSA groups. Obesity was not an independent predictor of mortality in any group.

**Conclusions:**

This study showed obesity was not found to be a predictor of mortality for any burn size but did influence extended ICU LOS, hospital LOS and total hospital costs. Standardizing the data dictionary and adding obesity-related fields may greatly strengthen the role of the NBR in obesity-related burn research.

**Applicability of Research to Practice:**

Utilizing the NBR and understanding its limitations can help to improve this database and better understand the obese burn patient population.

